# Genome-Wide Identification, Phylogeny, and Expression Profile of the Dmrt (Doublesex and Mab-3 Related Transcription Factor) Gene Family in Channel Catfish (*Ictalurus punctatus*)

**DOI:** 10.3389/fgene.2022.891204

**Published:** 2022-04-28

**Authors:** Siqi Xu, Shiyong Zhang, Wenping Zhang, Hongyan Liu, Minghua Wang, Liqiang Zhong, Wenji Bian, Xiaohui Chen

**Affiliations:** ^1^ National Genetic Breeding Center of Channel Catfish, Freshwater Fisheries Research Institute of Jiangsu Province, Nanjing, China; ^2^ College of Marine Science and Fisheries, Jiangsu Ocean University, Lianyungang, China; ^3^ The Jiangsu Provincial Platform for Conservation and Utilization of Agricultural Germplasm, Nanjing, China

**Keywords:** Dmrt gene family, sex reversal, channel catfish, sex determination, animal reproduction

## Abstract

The *Dmrt* (Doublesex and Mab-3 related transcription factor) gene family is a class of crucial transcription factors characterized by a conserved DM domain related to sex determination and differentiation, which has been systematically described in various teleost fish, but less in channel catfish (*Ictalurus punctatus*), an important global aquaculture species in the US and China. In this study, seven *Dmrt* genes from channel catfish genome were identified and analyzed using bioinformatics methods. Seven *IpDmrt* genes were distributed unevenly across five chromosomes. Synteny analysis revealed that *Dmrt1*, *Dmrt2a*, *Dmrt2b*, *Dmrt3*, *Dmrt4*, and *Dmrt5* were relatively conserved in teleost fish. Tissue distribution analysis showed that *IpDmrt1*, *IpDmrt2b*, *IpDmrt5*, and *IpDmrt6* exhibited sexually dimorphic expression patterns and, among them, *IpDmrt1* and *IpDmrt6* had high expression levels in the testes, while *IpDmrt2b* and *IpDmrt5* had more significant expression levels in the ovaries than in other tissues. After 17β-estradiol treatment, *IpDmrt2b* and *IpDmrt5* were significantly up regulated, while the expression of *IpDmrt1* and *IpDmrt6* was significantly repressed in XY channel catfish ovaries compared with XX channel catfish ovaries. The present study provides a comprehensive insight into the *Dmrt* gene family of channel catfish. The results suggest that *IpDmrt1* and *IpDmrt6* may play an important role in testis differentiation/development, while *IpDmrt2b* and *IpDmrt5* are critical in ovary development in this species.

## Introduction

Sex determination (SD) in vertebrates is a complicated physiological process, affected by both environmental and genetic factors (Shen and Wang, 2014). A diverse array of SD mechanisms, including environmental SD (ESD), and genetic SD (GSD); or a combination of both, have been identified among these animals (Ortega-Recalde et al., 2020). During the evolution of organisms from lower to higher forms, genetic factors gradually increased, while environmental factors weakened. With the evolution of mammals, the former completely replaced the impact of the latter on gonadal sex differentiation (Zhou et al., 2004). In lower vertebrates such as reptiles, amphibians, fish due to the relatively low degree of evolution, as well as the lack of an obvious difference in sex chromosomes, environmental factors, such as temperature, social status, or pH, had a certain impact on early sex differentiation, and could even induce sexual reversal and change the sex differentiation pathway (She and Yang, 2017).

The reproductive modes of teleosts are divided into gonochorism and hermaphroditism (Nagahama et al., 2021). In nature, most teleosts are gonochoristic, exhibiting two differentiated forms, namely, male and female. However, hermaphroditism is also common among teleosts, and in this mode both testes and ovaries exist either simultaneously (e.g., in coral reef fish) (Godwin et al., 2000) or sequentially (e.g., in rice field eel) (Xiao et al., 2010). The primordial gonad in gonochoristic fish can develop either as a testis or an ovary, thus the direction of gonadal sex differentiation depends on their sex-determining genes present in the genome, or on environment conditions. To date, several master sex-determining genes have been characterized in teleosts, such as *Dmy* from Japanese medaka (*Oryzias latipes*) (Matsuda et al., 2002), *Gsdf* from Philippine medaka (*Oryzias luzonensis*) (Myosho et al., 2012), *Amh* from Patagonian pejerrey (*Odontesthes hatcheri*) (Hattori et al., 2012), *Amhr2* from Japanese pufferfish (*Fugu rubripes*) (Kamiya et al., 2012), *Sox3* from brackish medaka (*Oryzias dancena*) (Takehana et al., 2014), and *Dmrt1* from half-smooth tongue sole (*Cynoglossus semilaevis*) (Cui et al., 2017).

Although sex-determining genes vary in teleost fish, the *Dmrt* (Doublesex and Mab-3 related transcription factor) gene family is thought to be associated with sex determination and differentiation, embryo and gonadal development, as well as muscle growth, in vertebrates ranging from fish to tetrapods. This gene family belongs to the transforming growth factor-beta superfamily, which contains one or several classical DNA-binding domains. There are at least five *Dmrt* genes (*Dmrt1*, *Dmrt2a*, *Dmrt2b*, *Dmrt3* and *Dmrt5*) in teleost fish, among which *Dmrt1* is the major male-biased expression gene (Herpin and Schartl, 2011; Chen et al., 2014) participating directly in sex determination and differentiation through the activation of the *gsdf*-promotor and inhibition of the *Cyp19a1a*-promotor alone in both XY and ZZ male teleosts (Han et al., 2021).

Channel catfish (*Ictalurus punctatus*) is one of the most important freshwater aquaculture fish in the US and China (Chen et al., 2016). Due to its strong environmental adaptability, superior muscle quality, lower number of intermuscular spines, and easy meat processing characteristics, it is currently bred worldwide (Bao et al., 2019). The annual production of channel catfish has been stable at more than 4 × 10^9^ kg in China (Zhong et al., 2021). At present, the *Dmrt* gene family has been systematically described in various teleost fish, but less so in channel catfish. The aim of this study was to provide a comprehensive insight into this important aquaculture species by identifying its *Dmrt* gene family members and analyzing their physical and chemical properties, gene phylogeny, as well as gene expression patterns.

## Materials and Methods

### Fish Sampling

The channel catfish fries used in the present study were produced at the National Genetic Breeding Center of Channel Catfish (Yangzhong, Jiangsu, China). Artificial compound feed containing 17β-estradiol (17β-E_2_, 60 μg/g) was selected as a vector to induce the sexual reversal of male fish by feeding catfish larvae continuously for 27 days. The dosage of 17β-E_2_ in the compound feed was 60 μg/g. The gonads of sex-reversed and normal female catfish at 60 dahs were identified by histological section and sex-linkage molecular marker (Zhang et al., 2019; Pan et al., 2022), respectively.

Furthermore, the ovary tissues of the two catfish types were dissected from three individuals based on the identification results. At the same time, five tissues, namely, liver, brain, kidney, head kidney, and testes/ovaries, were also dissected from three male and three female individuals. All tissue samples were preserved in the RNA Keeper Tissue Stabilizer (Vazyme, China) and were stored at -20°C until RNA extraction. During sampling, the experimental fish were anesthetized using 0.1% tricaine methanesulfonate (MS-222) (Merck, Germany). All animal handling was carried out in accordance with the ethical guidelines and protocols of the Freshwater Fisheries Research Institute of Jiangsu Province.

### Identification of Dmrt Family Genes

Candidate *IpDmrt* family genes were identified on the channel catfish genome using the BLAST search program based on the conserved DM domain (Pfam 00,751) with amino acids. The identified candidates and their amino acid sequences were downloaded from the channel catfish genome database stored in the NCBI database (https://www.ncbi.nlm.nih.gov/). The genes were further confirmed to contain the conserved DM domains by scanning their sequences through the Pfam (http://pfam.sanger.ac.uk/search), SMART (http://smart.embl-heidelberg.de/), and InterPro (http://www.ebi.ac.uk/interpro/) online databases.

The total RNA of sampled tissues was isolated using the RNeasy Mini Kit (Qiagen, Germany). The cDNA was subjected to reverse transcription using the PrimeScript™ first Strand cDNA Synthesis Kit (TaKaRa, Dalian, China) according to the manufacturer’s instructions. According to the DNA sequences of channel catfish *Dmrt* genes, we designed seven pairs of primers (Supplementary Table S1) to clone their full-length cDNA sequences. The PCR conditions were as follows: 94°C for 30 s, gene-specific annealing for 30 s and elongation stage at 72°C for 60 s, a total of 34 cycles. The target products were purified using the universal DNA Purification Kit (Tiangen, Beijing, China), and then sequenced by the Sangon Biotech Co., Ltd. (Shanghai, China).

Basic information related to the *IpDmrt* genes, including the number of introns and exons, coding sequence length, and chromosome location, were extracted from the channel catfish genome database. The physicochemical characteristics of IpDmrt proteins, including the theoretical molecular weight (kDa), lengths, isoelectric points (pI), and grand average of hydropathicity (GRAVY), were evaluated using the online ExPASY tool (https://web.expasy.org/protparam/). The intron and exon positions, and untranslated regions were visualized using the Gene Structure Display Server 2.0 (http://gsds.gao-lab.org/).

### Multiple Alignment and Phylogenetic Analysis

Multiple sequence alignments of the IpDmrt proteins were performed and visualized using the T-COFFEE (tcoffee.crg.cat/apps/tcoffee/index.html) and EsPript 3.0 (https://espript.ibcp.fr/ESPript/ESPript) online software, respectively. The amino acid sequences of Dmrt proteins from human (*Homo sapiens*), chicken (*Gallus*), African clawed frog (*Xenopus laevis*), zebrafish (*Danio rerio*), large yellow croaker (*Larimichthys crocea*), Nile tilapia (*Oreochromis niloticus*), Japanese medaka, Japanese pufferfish, and channel catfish were selected to construct the phylogenetic tree in MEGA7 using the neighbor-joining method. The Jones-Taylor-Thornton (JTT) algorithm + Nearest-Neighbor-Interchange (NNI) distance were selected to automatically generate the initial tree (Default - NJ/BioNJ) model and evaluate the phylogenetic tree. The *Dmrt* genes were named using an “*Ip*” prefix and were numbered in ascending order based on the results of the phylogenetic analysis. The name of Dmrt protein sequences showed in Supplementary Table S2.

### Chromosomal Distribution and Synteny Analysis

In order to examine the extent of preservation of genomic neighborhoods between *IpDmrt* genes and the potential counterparts of other species, such as electric eel (*Electrophorus electricus*), Nile tilapia, zebrafish, and Atlantic cod (*Gadus morhua*), the Synteny Database (http://teleost.cs.uoregon.edu/synteny_db/) and Genomicus Browser (http://www.dyogen.ens.fr/genomicus-63.01/cgi-bin/search.pl) were used as the main visualization tools. To perform the synteny analysis, the channel catfish orthologs of the *Dmrt* genes neighboring other teleosts’ genes were firstly examined at the chromosome level through the Synteny Database, and at the local neighborhood level using the Genomicus Browser, which provides detailed gene information related to each teleost gene’s orthologs on an individual chromosome. In the synteny analyses of *Dmrt* genes, the channel catfish neighboring genes as follows: *Fbrsl1, Ap1b1, Fbxo21, Fbxw8, Abca2, Adamts3, Dmrt2a, Dmrt3, Dmrt1*, and *Adgrv1* on Chr22; *Abl2, Bend5, Dmrt5, Calr, Abhd17 ab, Tmem47, Tab3, Nexn, Armh1, Multh,* and *Acbd6* on Chr5; *Irrc40, Irrc7, Insl5a, Ak4, Jak1, Foxd3, Kank4, Cyldl, Adcyaplr1b*, and *Dmrt2b* on Chr11; and *Ndrg2, Arhgef40, Casq1b, Ipcat4, Khnyn, Drap1, Rela, Elavl2, Dmrt4, Rbpms*, and *Alpk1* on Chr29.

### Protein Interaction Network Analysis

The purpose of protein interaction network analysis was to examine the relationship between Dmrt and other genes and explore their potential functions. The protein interaction networks of the *IpDmrt* gene family were constructed using the STRING online software (https://www.string-db.org).

### Quantitative Real-Time (qRT)-PCR Analysis

Gene-specific primers (listed in Supplementary Table S3) were designed using Primer Premier 5.0 software and were synthesized by Sangon Biotech Co., Ltd (Shanghai, China). The *α-tubulin* (Zhang et al., 2020) gene was employed as an internal reference gene. The qRT-PCR reaction system contained 12.5 μL of SYBR green (TaKaRa, Dalian, China), 1 μL of gene-specific primers (1.0 μM), 1 μL of cDNA, and 8.5 μL of DEPC water, reaching a total reaction volume of 25 μL. The PCR conditions were as follows: 95°C for 30 s, followed by 40 cycles of 15 s at 95°C and 30 s at 57°C; 95°C for 15 s, 60°C for 15 s, and 95°C for 15 s. Relative mRNA expression levels were determined using the 2^−△△ CT^ method (Livak and Schmittgen, 2001). Finally, the statistical significance was calculated in R v. 4.0.5. Statistically significant differences were set at *p* < 0.05.

## Results

### Identification and Annotation of *Dmrt* Genes in Channel Catfish

A total of seven *Dmrt* genes (*IpDmrt1*, *IpDmrt2a*, *IpDmrt2b*, *IpDmrt3*, *IpDmrt4*, *IpDmrt5*, and *IpDmrt6*) were identified in the channel catfish genome, and were named based on the nomenclature of teleost *Dmrt* genes and the results of phylogenetic analyses. These gene numbers were relatively consistent compared to that previously reported in other fish species, such as large yellow croaker (Wan et al., 2020), while it was less than that reported for grass carp (*Ctenopharyngodon idella*) (Chen et al., 2019) and rainbow trout (*Oncorhynchus mykiss*) (Wang et al., 2014), which experienced four rounds of whole genome duplication during evolution. The exon-intron structure analysis revealed that the *IpDmrt* gene family contained two to five exons; in particularly, *IpDmrt1* had five exons; *IpDmrt2a* and *IpDmrt6* had four; *IpDmrt2b* had three; and *IpDmrt3*, *IpDmrt4*, and *IpDmrt5* had two. The length of IpDmrt proteins ranged from 282 to 507 amino acids, with an average molecular weight of 42.30062 kDa. The average pI of the IpDmrt protein family was 8.13; with the highest and lowest points reaching 9.36 and 5.91, respectively. Only one basic protein, IpDmrt3, was detected, while the others were all acidic proteins. The calculated grand average of the GRAVY values of all *IpDmrt* genes was -0.2835 to -0.928, indicating that they were hydrophobic in nature (Table1).

**TABLE 1:** Basic physical and chemical properties of *IpDmrt* genes.

Gene	Amina Acids number (aa)	Exon Count	MW (kDa)	PI	GRAVY
IpDmrt1	293	5	31.93265	7.48	−0.7445
IpDmrt2a	507	4	55.71698	8.90	−0.9165
IpDmrt2b	370	3	41.62255	9.36	−0.333
IpDmrt3	435	2	47.70946	5.91	−0.2835
IpDmrt4	385	2	41.67151	9.23	−0.411
IpDmrt5	432	2	46.53243	7.14	−0.661
IpDmrt6	282	4	30.95758	8.87	−0.928

**FIGURE 1:**
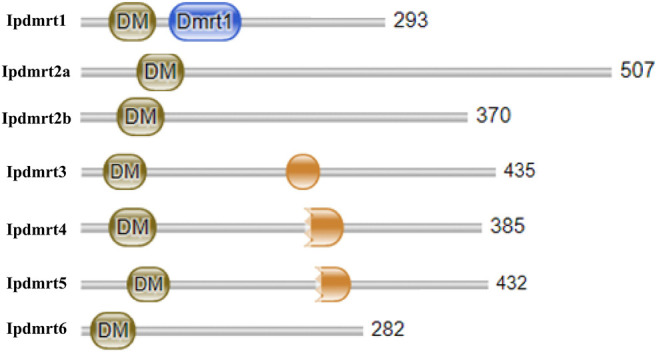
Schematic diagram of the construction of seven IpDmrt proteins. Image (a) shows the schematic diagram of seven IpDmrt protein domains; all the identified proteins present a conserved DM domain, and IpDmrt1 has an additional DMRT-1 domain.

Each IpDmrt protein had a characteristic conserved DM domain (Pfam 00,751), and only protein IpDmrt1 had an additional Dmrt1 domain, while a DMA domain was detected in IpDmrt3, IpDmrt4, and IpDmrt5 (Figure 1).

### Multiple Alignment and Phylogenetic Analysis

**FIGURE 2:**
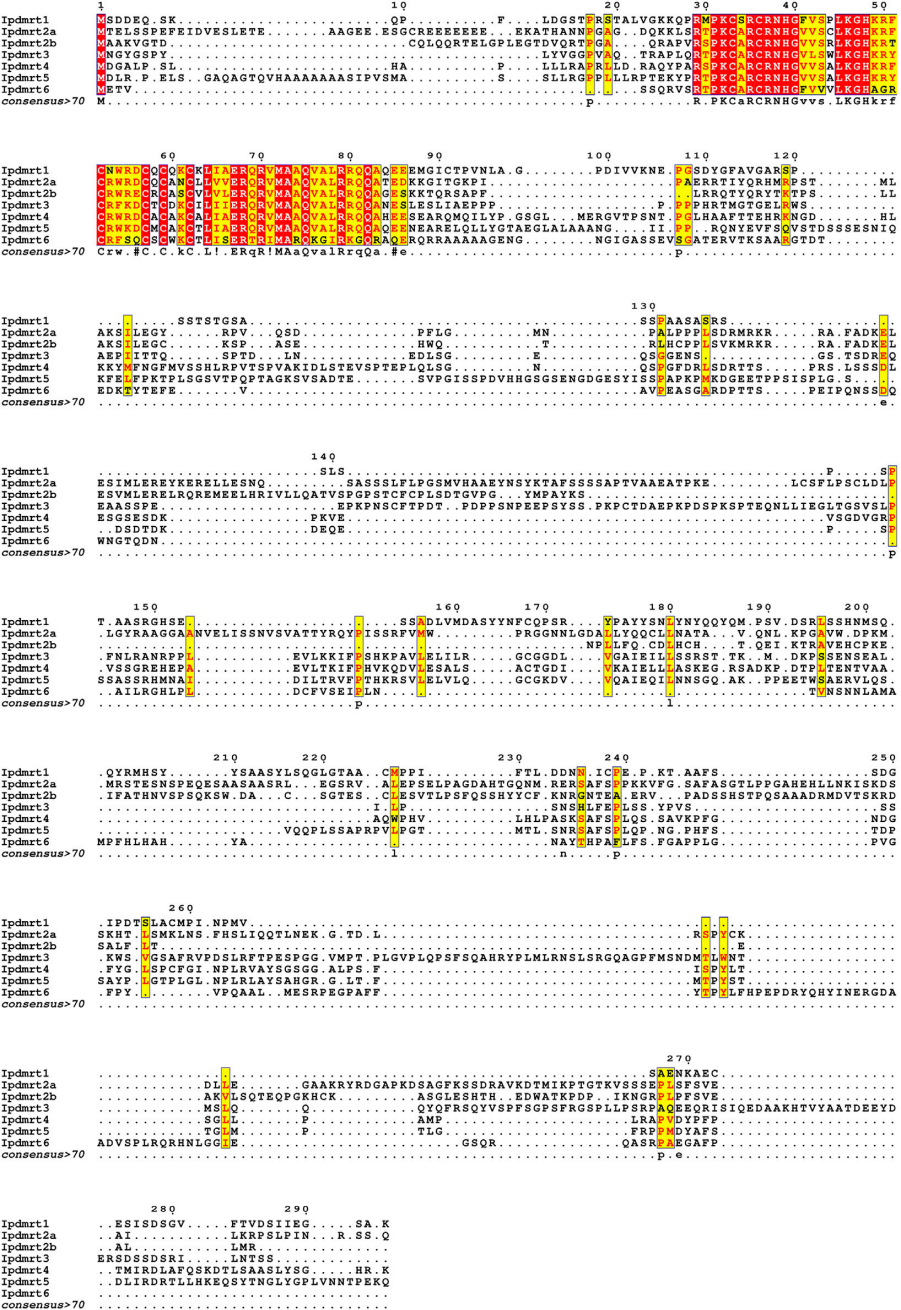
Multiple sequence alignments of Dmrt proteins. The alignments were performed using the T-COFFEE online software, and were visualized in EsPript v. 3.0 (available online). The partial protein sequence in the red box represents the DM domain, which has a higher conservation, while the protein sequence outside it has a lower level of identity.

The multiple alignment of IpDmrt protein sequences revealed the presence of a conserved DM domain at the N-terminal (Figure 2), while outside the DM domain there was an extremely lower conservation. Sequence identity analysis of DM domain showed that IpDmrt1 shared a 75% identity with its homologs from IpDmrt2a (74.58%) and IpDmrt3 (66.10%), which were located on the same chromosome (Chr 22). However, IpDmrt1 shared a higher identity with its counterparts from IpDmrt5 (82.76%). Although IpDmrt4 and IpDmrt5 were on the different chromosome, they shared the highest percentage of identity (89.66%) compared to all chromosomes (Table 2).

**TABLE 2:** Identity of the amino acid sequences of *IpDmrt* genes.

Gene	IpDmrt1 (%)	IpDmrt2a (%)	IpDmrt2b (%)	IpDmrt3 (%)	IpDmrt4 (%)	IpDmrt5 (%)
IpDmrt6	52.54	54.24	56.36	58.18	57.63	64.41
IpDmrt5	81.03	81.36	77.59	77.97	89.66	
IpDmrt4	82.76	81.36	81.03	72.88		
IpDmrt3	66.10	72.88	71.19			
IpDmrt2b	72.41	79.66				
IpDmrt2a	74.58					

**FIGURE 3:**
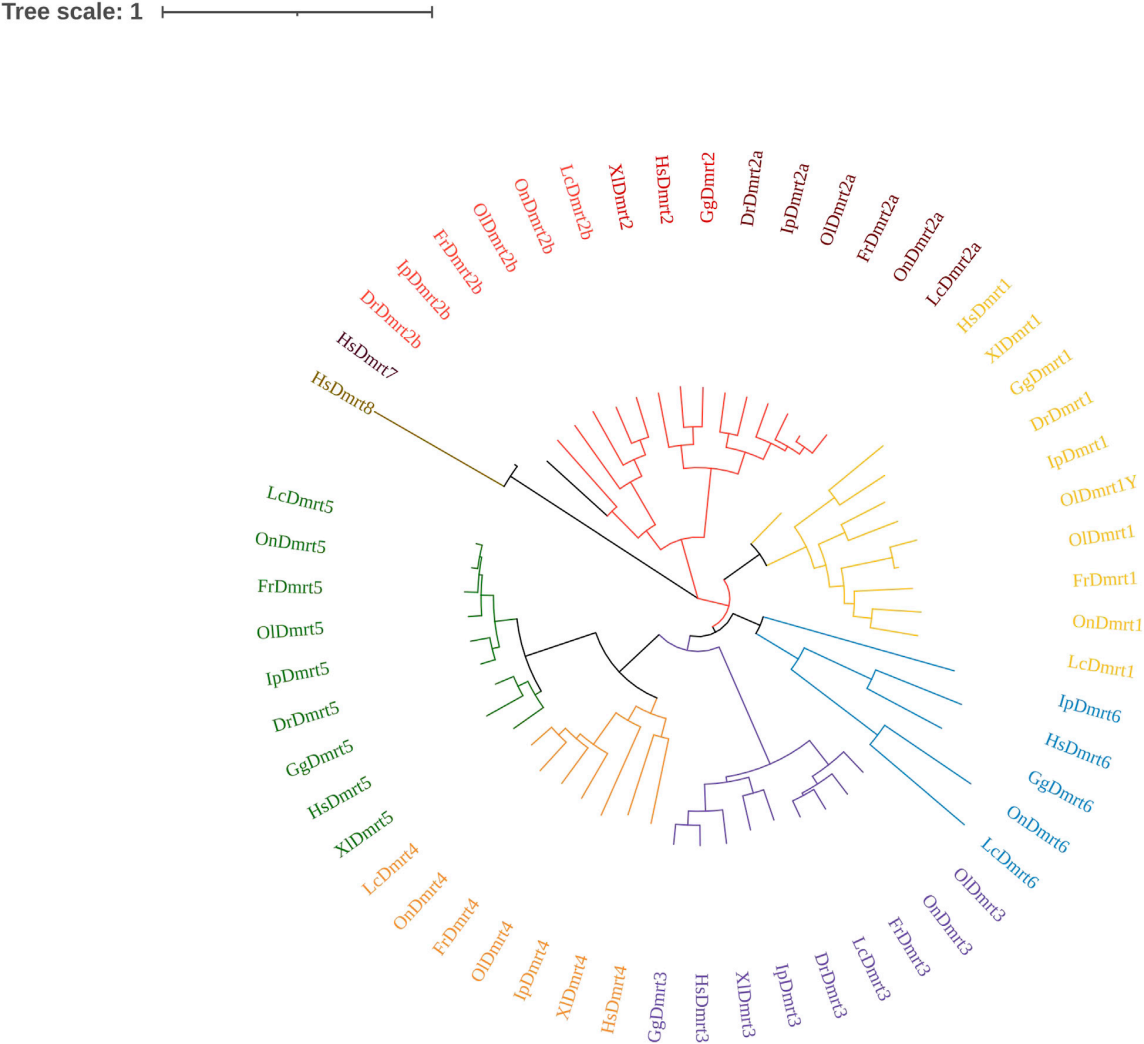
Phylogenetic tree analysis of *Dmrt* gene family members from human, chicken, African clawed frog, zebrafish, large yellow croaker, Nile tilapia, Japanese pufferfish, and channel catfish. The phylogenetic tree was constructed in MEGA7, using the neighbor-joining method. The GenBank accession numbers of the sequences used are listed in the Supplementary Table S1.

To analyze the phylogenetic relationships among the *IpDmrt* gene family members, a phylogenetic tree was generated using 57 Dmrt protein sequences from various vertebrate species, ranging from fish to mammals, based on the maximum likelihood method. As shown in Figure 3, *Dmrt1*, *Dmrt2a*, *Dmrt3*, and *Dmrt5* were found in all five teleost species. As expected, *Dmrt* genes were clustered into six subfamilies, which is consistent with previous studies (Dong et al., 2020; Kikkawa and Osumi, 2021), and each *Dmrt* gene branched along with its counterparts from different vertebrate species. Within each subfamily, the phylogenetic topology could be further divided into two subgroups: tetrapods and teleosts. *Dmrt2* experienced teleost-specific whole genome duplication during evolution, and at least two copies (*Dmrt2a* and *Dmrt2b*) were observed in teleosts. However, *Dmrt7* and *Dmrt8* were lost in teleosts, and are only present in the mammal genome (Veith et al., 2006; Date et al., 2012).

### Synteny Analysis

Gene duplication is an important process in species evolution in terms of gene expansion and functional diversity. The aim of synteny analysis is to show whether genes are conserved during evolution in teleosts. Conservative regions can be clearly identified by observing the distribution and permutation of genes on chromosomes. In this study, in order to investigate the preservation extent of the *Dmrt* genes among teleosts, the distribution and permutation on the corresponding chromosomes of these genes were compared, as well as their neighboring genes from channel catfish, zebrafish, electric eel, Nile tilapia, and Atlantic cod. Seven *IpDmrt* genes were unevenly distributed on five chromosomes by chromosomal location analysis. Specifically, *IpDmrt2b*, *IpDmrt4*, *IpDmrt5*, and *IpDmrt6* were located on Chr11, Chr29, Chr5, and Chr7, respectively; while *IpDmrt1*, *IpDmrt2a*, and *IpDmrt3* were located on the same chromosome (Chr22).

**FIGURE 4:**
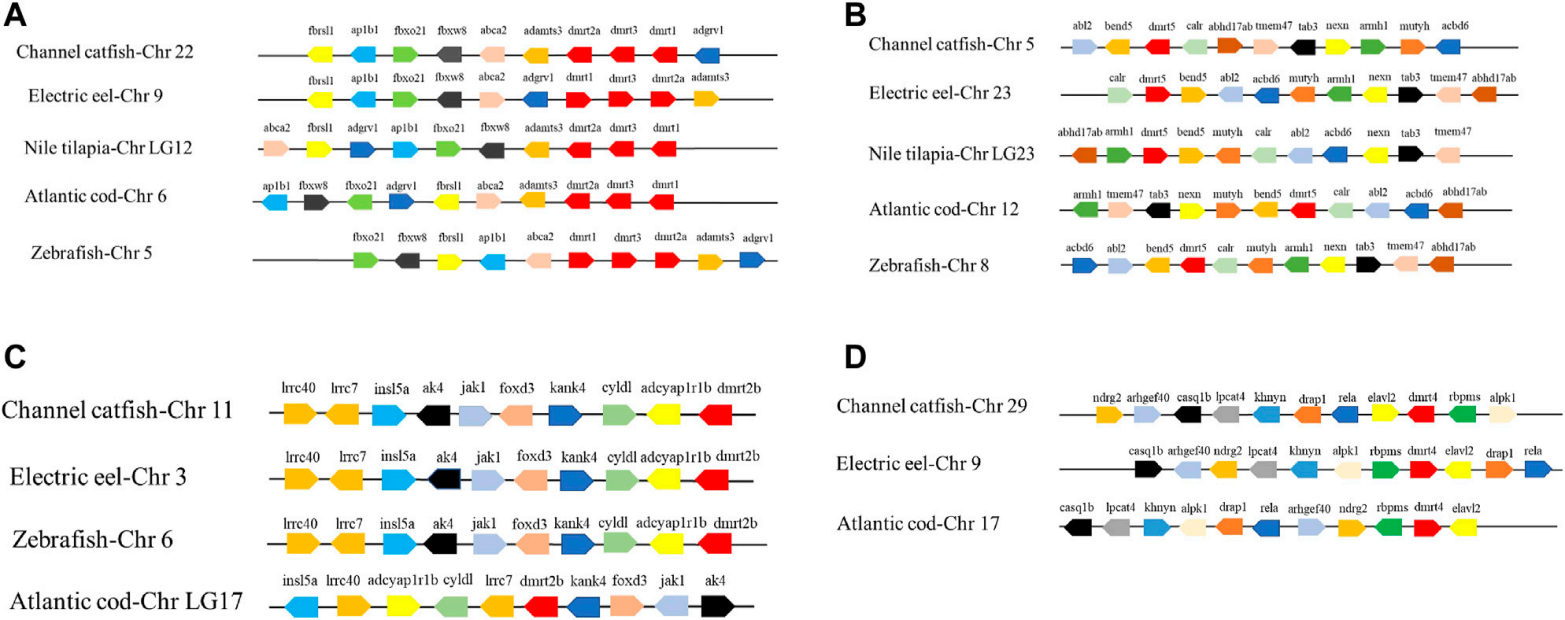
Synteny analysis of *Dmrt1*, *Dmrt2*, *Dmrt3* **(A)**, *Dmrt5* **(B)**, *Dmrt2b* **(C)**, *Dmrt4* **(D)** and their adjacent genes in electric eel, Nile tilapia, Atlantic cod, and zebrafish. Direction of the arrows indicates gene orientation.

Syntenic analyses revealed that the *Dmrt1-Dmrt2a-Dmrt3* syntenic block was highly conserved in teleosts (Figure 4A). Interestingly, *Dmrt5* and neighboring genes were not completely conserved, showing differences in replication direction and location (Figure 4B), which indicated that this region experienced genome arrangement during evolution. On the contrary, the most conservative syntenic block, *Irrc40-Irrc7-Insl5a-Ak4-Jak1-Foxd3-Kank4-Cyldl-Adcyaplr1b-Dmrt2b*, was detected in channel catfish, electric eel, and zebrafish, despite the location and copy direction of these genes being different in Atlantic cod (Figure 4C). It was also found that *Dmrt4* is preserved only in channel catfish, electric eel, and Atlantic cod, while it was lost in zebrafish and Nile tilapia (Figure 4D).

### Protein Interaction Network Analysis of IpDmrt

**FIGURE 5:**
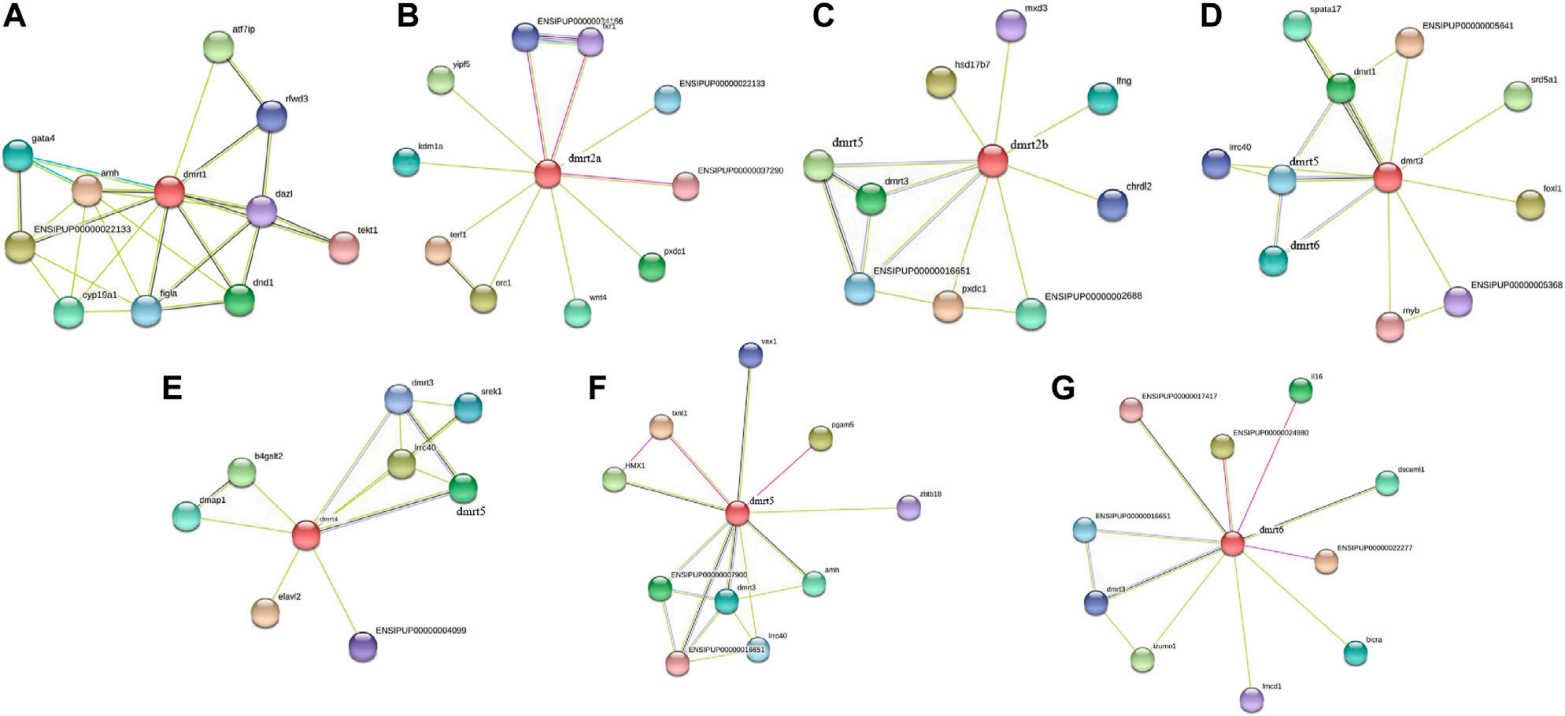
Interaction network analysis of IpDmrt proteins. Images **(A)**, **(B)**, **(C)**, **(D)**, **(E)**, **(F)**, and **(G)** show the protein interaction network of IpDmrt1, IpDmrt2a, IpDmrt2b, IpDmrt3, IpDmrt4, IpDmrt5, and IpDmrt6, respectively.

The interaction network of channel catfish Dmrt proteins was constructed using the STRING database. Dmrt1 closely interacted not only with the Amh protein, which is thought to be associated with male sex determination, but also with Cyp19a1, a key downstream gene of female sex regulation (Figure 5A). Both Dmrt2a and Dmrt2b were related to Pxdc1 in the protein interaction network; in addition, Dmrt2a was also associated with Wnt4 (Figures 5B, C); Figures 5D–F showed that there was protein interaction among Dmrt3, Dmrt4, and Dmrt5. Moreover, Dmrt3 was demonstrated to interact with Foxl1, which was also compactly linked with Dmrt6 in the protein interaction network (Figure 5G). This analysis revealed that most genes interacting with *Dmrt* genes have functions in sex differentiation and gonad development, contributing to understand the mechanism/dynamics of more genes related to sex determination and differentiation in channel catfish and even in teleosts.

### Expression Profiling of *IpDmrt* Genes in Different Tissues

**FIGURE 6:**
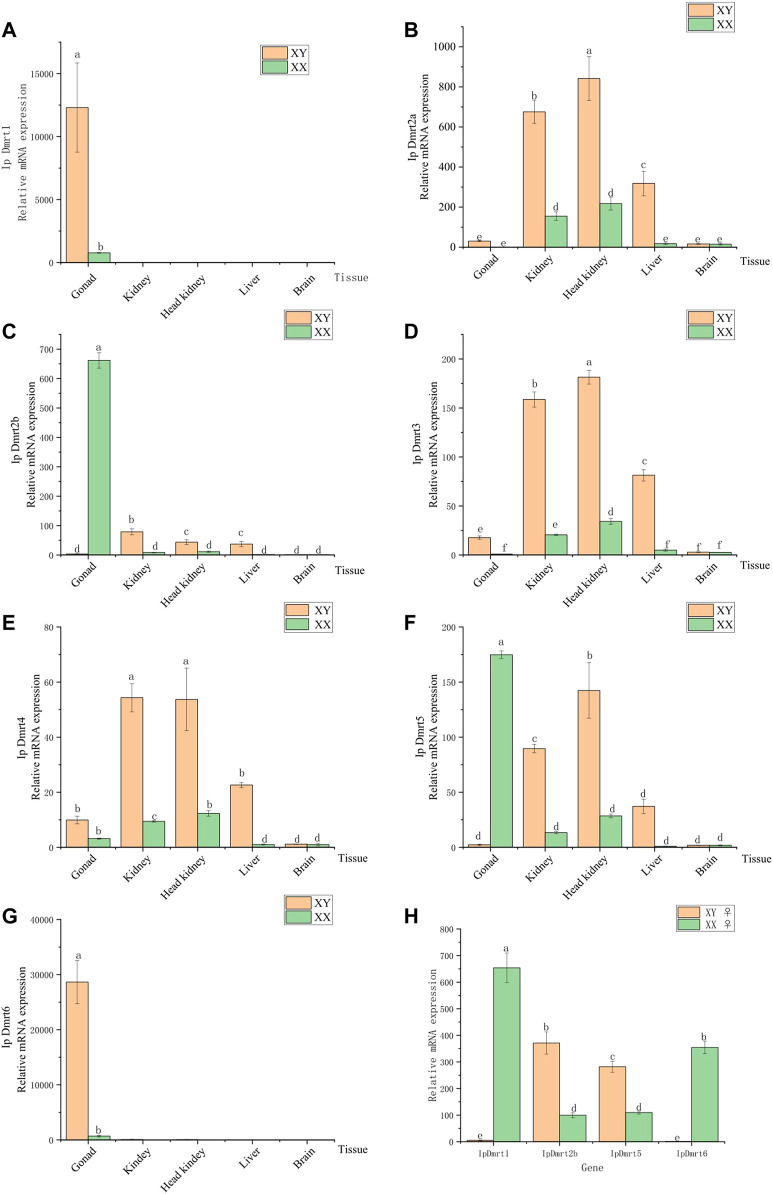
Results of the qRT-PCR assay for channel catfish *Dmrt* genes in testes and ovaries. The gene expression level was normalized to the *α-tubulin* transcript. Images **(A)**, **(B)**, **(C)**, **(D)**, **(E)**, **(F)**, and **(G)**, show the *Dmrt* genes expression level in gonad, kidney, head kidney, liver, and brain. Images **(H)** show the *Dmrt1*, *Dmrt2b*, *Dmrt5* and *Dmrt6* genes expression level in gonad which are female (XX) and female (XY). In each image, different letters represent significant differences among lines (*p* < 0.05).

To study the spatiotemporal specific expression of *IpDmrt* genes, their expression profiles were investigated in five tissues obtained from female and male channel catfish, namely the brain, head kidney, kidney, liver, and ovaries or testes using qRT-PCR. Subsequently, the mRNA expression of *IpDmrt* genes in the ovary tissues of sex-reversed individuals were also explored. The results indicated that *IpDmrt1* and *IpDmrt6* exhibited a sexually dimorphic expression pattern with higher expression levels in the testes than in the ovaries (*p* < 0.01, Figures 6A, G). *IpDmrt2a*, *IpDmrt3*, and *IpDmrt4* were highly expressed in the kidney, head kidney, and liver of male channel catfish (*p* < 0.05, Figures 6B, D, E). In addition, a sexually dimorphic expression pattern was also detected in *IpDmrt2b* and *IpDmrt5*, which presented more significant expression levels in the ovaries than in other tissues (*p* < 0.05, Figures 6C, F). Also, a relatively high expression level of *Dmrt5* was also detected in the kidney, head kidney, and liver of male channel catfish (*p* < 0.05, Figure 6F).

The qRT-PCR assay was performed to verify the response expression of *IpDmrt* genes during sex reversal. The expression of *IpDmrt2a*, *IpDmrt3*, and *IpDmrt4* was not carried out because a sexually dimorphic expression pattern was not observed. Compared with XX channel catfish, *IpDmrt2b* and *IpDmrt5* were significantly up-regulated in the ovaries of XY individuals, while *IpDmrt1* and *IpDmrt6* were significantly down-regulated. The results indicate that these *Dmrt* gene family members may play an important role during sex reversal in XY channel catfish.

## Discussion

Although it has been reported that the *Dmrt* family genes played vital roles in sex determination and differentiation in teleosts such as zebrafish (Graf et al., 2015; Webster et al., 2017), Chinese tongue sole (*Cynoglossus semilaevis*) (Cui et al., 2017; Feng et al., 2021), and spotted scat (*Scatophagus argus*) (Uma Farouk et al., 2018), to date, a thorough survey and research of the functions of *Dmrt* genes has not been conducted in channel catfish, even though its entire genome sequence has been available for several years (Chen et al., 2016; Ourth et al., 2017). In this study, a comprehensive genome-wide analysis identified a total of seven *Dmrt* genes in the channel catfish genome.

Previous reports have shown that the number of *Dmrt* genes range from five (zebrafish) to twelve (common carp), and this is correlated not only with genome size but also with genomic duplication rounds. For example, only five *Dmrt* genes were identified in Atlantic cod, which has a genome of 686 megabases (Mb) (Star et al., 2011; Johnsen and Andersen, 2012), while Atlantic salmon, whose genome size (2,970 Mb) is relatively large (Lien et al., 2016; Dong et al., 2020), has ten *Dmrt* genes. Both zebrafish and common carp (*Cyprinus carpio*), which belong to the order Cypriniformes, have relatively large genomes (>1,400 Mb) (Kerstin et al., 2013; De et al., 2018), but, as they underwent three and four rounds of genome replication, respectively, the former has only five *Dmrt* genes (Jocelyn, 2021), while the latter has 12. Compared with tetrapods, teleosts experienced three to four rounds of genome replication and seem to possess more *Dmrt* genes. However, *Dmrt7* and *Dmrt8* are present in mammals specifically, and only a couple of copy genes (*Dmrt2a* and *Dmrt2b*) are found in most 3R teleosts, which probably separated during the basal vertebrate genome duplication (2R).

The protein interaction network analysis showed that *Dmrt* genes have a close relationship with *Wnt4*, *Foxl*, *Amh*, and *Cyp19a1*. *Amh* (anti-Müllerian hormone) is considered necessary for female differentiation, because it regulates FSH to induce aromatase activity (Garg and Tal, 2016). Moreover, *Amh* is closely related to the development of spermatogonia by interacting with its *Amhr2* (anti Müllerian hormone receptor type 2) receptor in gonads (Wu et al., 2017). The *Foxl* gene family members have different functions, and *Foxl2* in particular is associated with ovary formation, as confirmed by numerous study (Perry et al., 2019). Another significant sex differentiation gene is *Wnt4*, which regulates the development of the Müllerian duct by controlling the secretion of sterol in mammals (Biason-Lauber and Konrad, 2008). Even if this structure is absent in teleosts, the genes that regulated its formation are present, and during this process they acquired a major and even more prominent role in sex determination (Adolfi et al., 2019). It has been proved that steroid hormones play crucial roles in the process of sex differentiation through indirect techniques, such as treatments with steroid hormones, steroid enzyme inhibitors, or steroid receptor antagonists (Paul-Prasanth et al., 2013). *Cyp19a1* is a key gene for the balancing of the secretion levels of endogenous steroid hormones, and regulated by several upstream genes, including *Dmrt1*, *Foxl2*, *Amh*, and *Wnt4*.

In the present study, the expression levels of *IpDmrt* genes were detected in several tissues based on the qRT-PCR assay. Among these genes, *IpDmrt1* and *IpDmrt6* had a higher expression in the testes, while *IpDmrt2b* and *IpDmrt5* had a higher expression in the ovaries, indicating that *IpDmrt1/IpDmrt6* and *IpDmrt2b/IpDmrt5* may have important regulatory roles in male and female sex differentiation/development, respectively. The expression levels of *IpDmrt1*, *IpDmrt2b*, *IpDmrt5*, and *IpDmrt6* in the ovaries of normal and XY channel catfish treated with 17β-estradiol also confirmed our hypothesis; in particular, *IpDmrt2b* and *IpDmrt5* were significantly up-regulated in XY channel catfish ovary, while *IpDmrt1* and *IpDmrt6* were significantly repressed. Numerous studies have proved that *Dmrt1* plays a dominant role in male sex differentiation in avians, turtles, frogs, and teleosts (Bellefroid et al., 2013). However, *Dmrt1* participates to antagonistic regulatory networks in association with *Foxl2* to maintain the male fate in mammals. *Dmrt6* is absent in most teleosts, such as zebrafish, medaka, fugu, and Atlantic salmon (Dong et al., 2020). This gene takes part in mammalian mitotic and meiotic developmental processes during spermatogenesis by repressing genes involved in spermatogonial differentiation and activating those required for meiotic prophase (Zhang T et al., 2014). It seems to have similar functions in bony fish; for example, *Dmrt6* knockout in tilapia resulted in fewer spermatocytes and produced a lower level of serum 11-ketotestosterone (Zhang X et al., 2014). Tetrapods have only one *Dmrt2* gene, while the majority of teleosts present at least two orthologous *Dmrt2* genes, *Dmrt2a* and *Dmrt2b*, of which the former is expressed in various tissues and promotes the transition of endochondral bone formation by linking *Sox9* and *Runx2* (Ono et al., 2021). *Dmrt2b* is different from *Dmrt2a*, *Dmrt2b* is significantly expressed in teleosts’ ovaries and is critical for their development (Liu et al., 2009). *Dmrt5* is required for the terminal differentiation of corticotropes and gonadotropes; this gene regulates corticotrope differentiation in the pituitary gland in a cell-autonomous manner, thereby determining gonadotrope numbers (Michaelidou et al., 2013).

## Data Availability

The original contributions presented in the study are included in the article/Supplementary Material, further inquiries can be directed to the corresponding authors.
