# Supplementary material for: Genome-Wide Identification, Phylogeny, and Expression Profile of the Dmrt (Doublesex and Mab-3 Related Transcription Factor) Gene Family in Channel Catfish (Ictalurus punctatus)
Source: Front Genet. 2022 Apr 28;13:891204. doi: 10.3389/fgene.2022.891204 (PMC9095985; doi:10.3389/fgene.2022.891204)
Supplement: Supplementary file 1 [file Table1.DOCX]

Supplementary Material

**Supplementary Materials: Table 1.** The primers used for cloning channel catfish *Dmrt* genes.

| **Gene** | **Primers** | | **Sequence (5’-3’)** | **Length (bp)** | **Tm (℃)** |
| --- | --- | --- | --- | --- | --- |
| IpDmrt1 | C-Dmrt1-F  C-Dmrt1-R | CGCGAGATGGAATGTTGAGGT  TCCCCTGTTGTCTGGATGAA | | 21  20 | 57.73  55.72 |
| IpDmrt2a | C-Dmrt2a-F  C-Dmrt2a-R | ACTGAGCTTTGGTGGACTGG  CGCATACAAACACAACCAGCA | | 20  21 | 57.67  56.28 |
| IpDmrt2b | C-Dmrt2b-F  C-Dmrt2b-R | AACGTCCTTCAGCGTCGTAG  AGTTTCAGAGTAACGCCTGC | | 20  20 | 57.40  55.12 |
| IpDmrt3 | C-Dmrt3-F  C-Dmrt3-R | CTCTTGTGGAACCGAGCGT  TCCGCCATGTCTCTTTTGTCAT | | 19  22 | 57.98  56.49 |
| IpDmrt4 | C-Dmrt4-F  C-Dmrt4-R | AGACATGGACGGCGCTCTT  TGACATTTCAAGGCAAGAGAGGA | | 19  23 | 59.73  55.90 |
| IpDmrt5 | C-Dmrt5-F  C-Dmrt5-R | CAATCGCTAGTCCATCGGGG  TTGCACCATTATCGCACGGA | | 20  20 | 58.49  57.24 |
| IpDmrt6 | C-Dmrt6-F  C-Dmrt6-R | TGAGTGATTGTTAGGTCGCTT  CACCAGTCCTCGCTTCTAATGA | | 21  20 | 53.41  56.58 |
|  |  |  | |  |  |

**Supplementary Materials: Table 2 The name of Dmrt protein sequences**

| Gene | Protein product accession NO. | Gene | Protein product accession NO. | Gene | Protein product accession NO. | Gene | Protein product accession NO. | Gene | Protein product accession NO. | Gene | Protein product accession NO. | Gene | Protein product accession NO. | Gene | Protein product accession NO. | Gene | Protein product accession NO. |
| --- | --- | --- | --- | --- | --- | --- | --- | --- | --- | --- | --- | --- | --- | --- | --- | --- | --- |
| Ip Dmrt 1 | XP 017308042.1 | Lc Dmrt1 | XP_027133892.1 | Dr Dmrt1 | NP_991324.2 | Xl Dmrt1 | NP_001089969.1 | Hs Dmrt1 | XP_016869864.1 | Gg Dmrt1 | XP_040511578.1 | Ol Dmrt1 | XP_004086499.1 | Fr Dmrt1 | NP_001033038.1 | On Dmrt1 | XP_013126365.1 |
| Ip Dmrt 2a | XP 017308058.1 | Lc Dmrt2a | XP_010734871.2 | Dr Dmrt2a | NP_571027.1 | Xl Dmrt2a | XP_018099312.1 | Hs Dmrt2a | NP_001374487.1 | Gg Dmrt2a | XP_003643035.3 | Ol Dmrt1Y | NP_001295953.1 | Fr Dmrt2a | NP_001033035.1 | On Dmrt2a | NP_001266696.1 |
| Ip Dmrt 2b | XP 017334613.1 | Lc Dmrt2b | XP_019120792.1 | Dr Dmrt2b | NP_001073445.1 | Xl Dmrt3 | XP_018114063.1 | Hs Dmrt3 | NP_067063.1 | Gg Dmrt3 | XP_429193.2 | Ol Dmrt2a | XP_023813898.1 | Fr Dmrt2b | XP_011613411.1 | On Dmrt2b | XP_005457139.1 |
| Ip Dmrt3 | XP 017308040.1 | Lc Dmrt3 | XP_010753534.3 | Dr Dmrt3 | NP_001005779.2 | Xl Dmrt4 | XP_018113157.1 | Hs Dmrt4 | NP_071443.2 | Gg Dmrt5 | XP_015146712.1 | Ol Dmrt2b | XP_004068077.2 | Fr Dmrt3 | NP_001033034.1 | On Dmrt3 | XP_003444527.2 |
| Ip Dmrt4 | XP 017316285.1 | Lc Dmrt4 | XP_010735509.1 | Dr Dmrt5 | NP_001007065.2 | Xl Dmrt5 | XP 018113897.1 | Hs Dmrt5 | NP_115486.1 | Gg Dmrt6 | XP_040560678.1 | Ol Dmrt3 | XP_023813900.1 | Fr Dmrt4 | XP_029695395.1 | On Dmrt4 | NP_001266410.1 |
| Ip Dmrt5 | XP 017323854.1 | Lc Dmrt5 | XP_027145940.1 |  |  |  |  | Hs Dmrt6 | NP_149056.1 |  |  | Ol Dmrt4 | XP_004079711.1 | Fr Dmrt5 | NP_001033039.1 | On Dmrt5 | XP_005479065.1 |
| Ip Dmrt6 | XP 017328630.1 | Lc Dmrt6 | XP_019113286.1 |  |  |  |  | Hs Dmrt7 | XP_016882612.1 |  |  | Ol Dmrt5 | XP_023810346.1 |  |  | On Dmrt6 | XP_003447317.1 |
|  |  |  |  |  |  |  |  | Hs Dmrt8 | NP_001074320.1 |  |  |  |  |  |  |  |  |

**Supplementary Materials: Table 3.** Details of the primers used for qRT-PCR in this study, including primer name, primer sequence, annealing temperature, and product length (bp) of the amplified *Dmrt* gene fragment and *α-tubulin*.

| **Gene** | **Primers** | **Sequence (5’-3’)** | **Length**  **(bp)** | **Tm**  **(℃)** | **Product length(bp)** |
| --- | --- | --- | --- | --- | --- |
| IpDmrt1 | Dmrt1-F  Dmrt1-R | GTGATTACGGCTTTGCGGTG  TAGCGGGAAGGCTGACAAAA | 20  20 | 57.39  56.71 | 193 |
| IpDmrt2a | Dmrt2a-F  Dmrt2a-R | TGCTGGAGCGGGAATACAAG  GGCAAGTCCAGACAGCTAGG | 20  20 | 57.75  58.42 | 205 |
| IpDmrt2b | Dmrt2b-F  Dmrt2b-R | GACGAGACTTCACTGCCCTC  ATCAGACAGCGGACAGAAGC | 20  20 | 58.24  57.63 | 190 |
| IpDmrt3 | Dmrt3-F  Dmrt3-R | TTCCGCTCGCAGTATGTCTC  TGCTTATGCGTTGCTCCTCT | 20  20 | 57.31  56.74 | 97 |
| IpDmrt4 | Dmrt4-F  Dmrt4-R | CAATCCACTGCGTGTTGCAT  GGATCATGGTGGGAAACGGA | 20  20 | 56.51  57.56 | 134 |
| IpDmrt5 | Dmrt5-F  Dmrt5-R | TCGGCGCTTAAAGGACACAA  CTCGCGCTTCGTTTTCTTCC | 20  20 | 57.10  57.26 | 145 |
| IpDmrt6 | Dmrt6-F  Dmrt6-R | CCGGAAGGTCCTGCTTTCTT  TCTGCAGGTCTGGATGCTTG | 20  20 | 57.72  57.73 | 170 |
| *α-tubulin* | α-tubulinF  α-tubulinR | AGCCATACAATTCCATCCTGACC  GCGGCAGATGTCGTAGATGG | 23  20 | 56.98  58.90 |  |
